# Analysis of the SNP rs3747333 and rs3747334 in NLGN4X gene in autism spectrum disorder: a meta-analysis

**DOI:** 10.1186/s12991-019-0227-5

**Published:** 2019-05-21

**Authors:** Hongli Sun, Ying Yang, Liyu Zhang, Haibin Wu, Huifang Zhang, Hui Li

**Affiliations:** 1 Shaanxi Institute of Pediatric Diseases, Affiliated Children Hospital of Xi’an Jiaotong University, Xi’an, Shaanxi People’s Republic of China; 2 Department of Cell Biology and Genetics, School of Basic Medical Sciences, Xi’an Jiaotong University Health Science Center, Xi’an, Shaanxi People’s Republic of China; 3 Department of PICU, Affiliated Children Hospital of Xi’an Jiaotong University, Xi’an, Shaanxi People’s Republic of China; 4 Department of Neonatology, Affiliated Children Hospital of Xi’an Jiaotong University, Xi’an, Shaanxi People’s Republic of China

**Keywords:** Autism spectrum disorder, NLGN4X, rs3747333, rs3747334, A meta-analysis

## Abstract

**Background:**

The SNP rs3747333 and rs3747334 in Neuroligin 4X (NLGN4X) gene have been demonstrated to be associated with the susceptibility to Autism spectrum disorder (ASDs; MIM 209850), but the results are inconsistent. Therefore, a meta-analysis of eligible studies reporting the association between rs3747333 and rs3747334 and ASD was carried out to enhance the reliability of published results.

**Methods:**

A systematic literature search was performed using PubMed, Web of Science, Cochrane Library to search English articles concerning the relation between rs3747333, rs3747334 and ASD up to Sep. 21th, 2017. Summary odds ratios (OR) and 95% confidence interval (CI) were used to evaluate the risk of rs3747333, rs3747334 in the ASD. The heterogeneity and publication bias of the eligible studies were also evaluated.

**Results:**

Six eligible studies involving 1284 subjects (735 patients and 549 healthy controls) were included in this meta-analysis. Overall, the results indicated that there was no significant risk elevation between rs3747333, rs3747334 variants and ASD (OR = 0.39, 95% CI 0.10–1.60). Furthermore, sensitivity analysis and publication bias analysis confirmed this result.

**Conclusions:**

In conclusion, our meta-analysis suggests that the rs3747333, rs3747334 in NLGN4X gene are not frequent causes of ASD.

**Electronic supplementary material:**

The online version of this article (10.1186/s12991-019-0227-5) contains supplementary material, which is available to authorized users.

## Background

### Introduction of autism spectrum disorder

Autism spectrum disorder (ASDs; MIM 209850) is a heterogeneous group of neurodevelopmental diseases defined by significant language delays, social, and communication deficits, and highly repetitive and stereotypic interest and/or behaviors [1]. The global prevalence of autism has risen to 6.2% [2]. Autism patients in China are estimated in the range of 0.6–1.8 million according to the WHO’s report. According to the latest report released by Autistica, people with ASD die on average 18 years before the general population [3]. ASD is a result of complex gene–environment interactions, with strong and clear genetic influences [4]. Although the etiologic and clinical heterogeneity are universally recognized, in practice, many studies still fail to take this into account. As of December, 2015, genetic studies have pointed to 791 potential candidate genes in ASD (https://gene.sfari.org/autdb/HG_Home.do), making it difficult to find common underlying pathogenic mechanisms.

### The risk of the rs3747333 and rs3747334 in the autism spectrum disorder

Neuroligin 4X (NLGN4X) is a member of neuroligins expressed in the postsynaptic neurons and mediate transsynaptic signaling by interacting their ligand, neurexins [5]. Several studies identified significant variants in the NLGN4X gene that were replicated in two or more independent samples and were associated with specific phenotypes of ASD [6–8]. However, other studies found no variants in the NLGN4X gene in autistic patients in their cohorts [9–11]. To date, many common single-nucleotide polymorphisms (SNPs) of NLGN4X such as rs72413786, rs2290487, rs7049300, rs3747333, rs3747334, have been studied in extensive research on ASD. Of these, the SNP rs3747333 and rs3747334 have received more favorable observations concerning on the ASD risk [11–16]. A case–control study of Chinese descendants found that the common SNPs (rs3747333 and rs3747334) in NLGN4X gene significantly associated with risk for autism [16], a previous work showed that seven boys were carriers of mutations at three SNPs including the synonymous SNP rs7049300, the non-synonymous SNP rs3747333 (L593) and the synonymous SNP rs3747334 (L593), but the co-occurrence of rs3747333C>T leading to a substitution of leucine to phenylalanine and rs3747334C>G entails a double substitution of CTC to TTG in codon 593 and thus protect the amino acid leucine [11]. However, a work from German showed that the polymorphisms rs 3747333 and rs 3747334 did not cause any amino acid substitutions in the total of the eight detected carriers [11]. Those inconsistencies could be attributed to the phenotypic heterogeneity of ASD and to relatively small sample sizes.

### Objective

Given the increasing attention directed to the inconsistencies results, to further clarify the inconclusive association between the two SNPs and ASD, we conducted a meta-analysis with available data published up to Sep. 21th, 2017.

## Materials and methods

### Literature search and selection strategy

A systematic literature search was conducted to collect studies relevant for the current meta-analysis. First, we searched three online databases (PubMed, Web of Science, Cochrane Library) for articles published in English on associations between the rs3747333, rs3747334 in NLGN4X and ASD up to Sep. 21th, 2017. Combinations of terms identifying the gene (rs3747333, rs3747334, NLGN4X, X-linked Neuroligin 4 gene) and terms identifying the phenotype of interest (autism, autism spectrum disorder, Asperger, childhood disintegrative disorder, pervasive developmental disorder) were entered as Medical Subject Heading terms and text words. After identifying a set of publications returned from each search, we collated the results and eliminated duplicates. We then reviewed the titles, abstracts, keywords and texts of all studies to exclude studies that were clearly irrelevant. Hand-searching was carried out to determine other potential eligible studies through scanning the references cited in the retrieved articles. Finally, we applied criteria to the set of studies to determine their inclusion or exclusion in the present meta-analysis. If the two reviewers (Hongli Sun and Ying Yang) disagreed, all the authors critically evaluated the studies to judge of the inclusion or exclusion of a certain study.

### Inclusion criteria

All the eligible articles meet the inclusion criteria:

1. The study was peer-reviewed to ensure high levels of methodological adequacy and to avoid the inevitable bias caused by dependence on investigators agreeing to provide data from unpublished studies, as suggested by the Cochrane Group [17].
2. The study was adopted as “Original Research”.
3. Assessment of the association between the rs3747333 or rs3747334 or both of them in NLGN4X and ASD.
4. Written in English.

### Data extraction

The two authors independently extracted the following data: (1) first author, (2) year of publication, (3) location, (4) predominant ethnicity of participants, (5) study design, (6) number of participants, (7) diagnostic criteria. When the study did not report sufficient data to calculate effect size, we contacted corresponding authors via e-mail to request additional information. If the contact with the author was not successful, we could not include the study in the meta-analysis.

### Statistical analysis

The association of the rs3747333, rs3747334 in NLGN4X and ASD was assessed by odds ratio (OR) with 95% confidence interval (CI). Subgroup (according to study design) analysis was evaluated by study type. Publication bias was considered present when *P*-value was less than 0.05. Sensitivity analysis was also carried out to evaluate the stability of the meta-analysis. Statistical heterogeneity among studies was evaluated by the Chi-square-based *Q*-test and the *I*^2^ index. *I*^2^ index < 50% and/or *P*-value > 0.10 for *Q*-test indicated a lack of heterogeneity among the studies. The fixed-effect model (the Mantel–Haenszel method) was utilized to calculate the pooled ORs when the heterogeneity was not significant among the studies. Otherwise, the random-effects model (the DerSimonian and Laird method) was employed. Publication bias was evaluated by the funnel plot. All statistical analyses were carried out with the STATA 12.0 (STATA Corp, College Station, Texas).

## Results

### Meta-analyses of the rs3747333, rs3747334 in NLGN4X and ASD

**Fig. 1:**
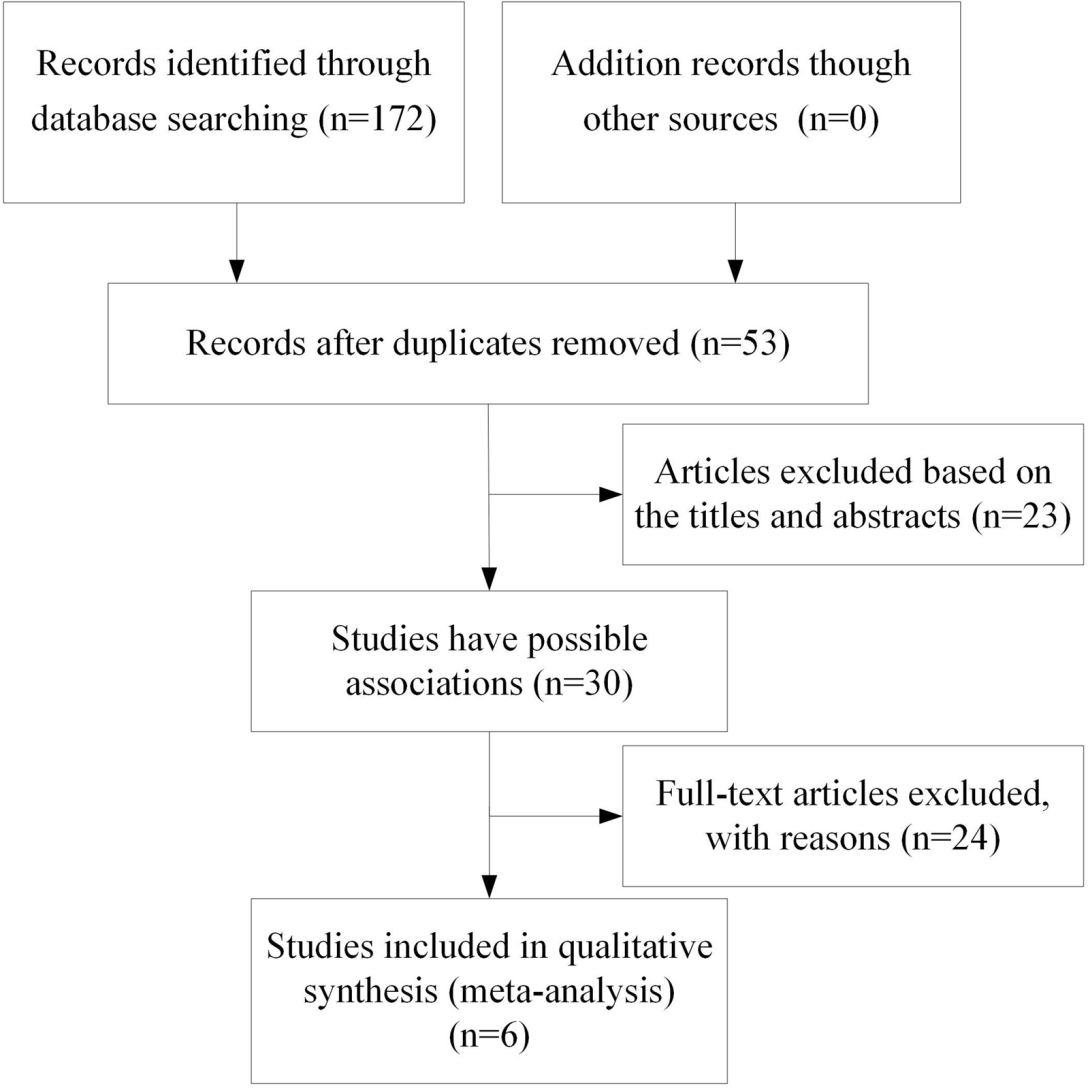
Flow diagram of this meta-analysis

After applying inclusion and exclusion criteria (see “Materials and methods”), data from 6 studies with 1284 subjects [9, 11, 14–16, 18] were included in the meta-analysis (Fig. 1). We assessed the association between the rs3747333, rs3747334, and ASD. The characteristics of the populations used for meta-analysis are shown in Table 1.

**Table 1:** Characteristics of all studies included in the meta-analysis NLGN4X SNP rs3747333 and rs3747334

Author, year	Ethnicity	Study design	Presence in patients/sex ratio		Presence in controls/sex ratio		Genotyping type	Diagnostic criteria	Nucleotide change
Mikhailov (2014)	Thai	Screening	9/143	5.8 M:1F	vs.dpSNP	Unknown	DHPLC^a^	DSM-IV^d^	c.1777 C>T c.17779C>G
Volaki (2013)	Greek	Screening	11/40	9 M:1F	vs.dpSNP	Unknown	PCR-DS^b^	DSM-IV	c.1777 C>T c.17779C>G
Wermter (2008)	German	Screening	7/107	20.4 M:1F	vs.dpSNP	Unknown	PCR–RFLP^c^	ICD-10^e^	c.1777 C>T c.17779C>G
Blasi (2006)	Italy	Screening	4/31	3.5 M:1F	vs.dpSNP	Unknown	PCR-RFLP^c^	DSM-IV	c.1777 C>T c.17779C>G
Gauthier (2005)	Quebec	Case–control	61/96	6.4 M:1F	56/96	8.6 M:1F	NA	DSM-IV, ADI-R^f^ and ADOS-G^g^	c.1777 C>T c.17779C>G
Xiaojuan (2014)	Chinese	Case–control	21/318	5.6 M:1F	4/453	3.0 M:1F	PCR-DS	DSM-IV and CARS^h^	c.1777 C>T c.17779C>G

^a^Denaturing high-performance liquid chromatography (DHPLC)

^b^Polymerase chain reaction-direct sequencing (PCR-DS)

^c^Polymerase chain reaction-based restriction fragment length polymorphisms (PCR-RFLP)

^d^Diagnostic and Statistical Manual of Mental Disorders, 4th ed. (DSM-IV)

^e^Tenth Revision of the International Classification of Diseases (ICD–10)

^f^Autism specific parent interview (ADI-R)

^g^Autism diagnostic observation scale (ADOS-G)

^h^Childhood autism rating scale (CARS)

**Fig. 2:**
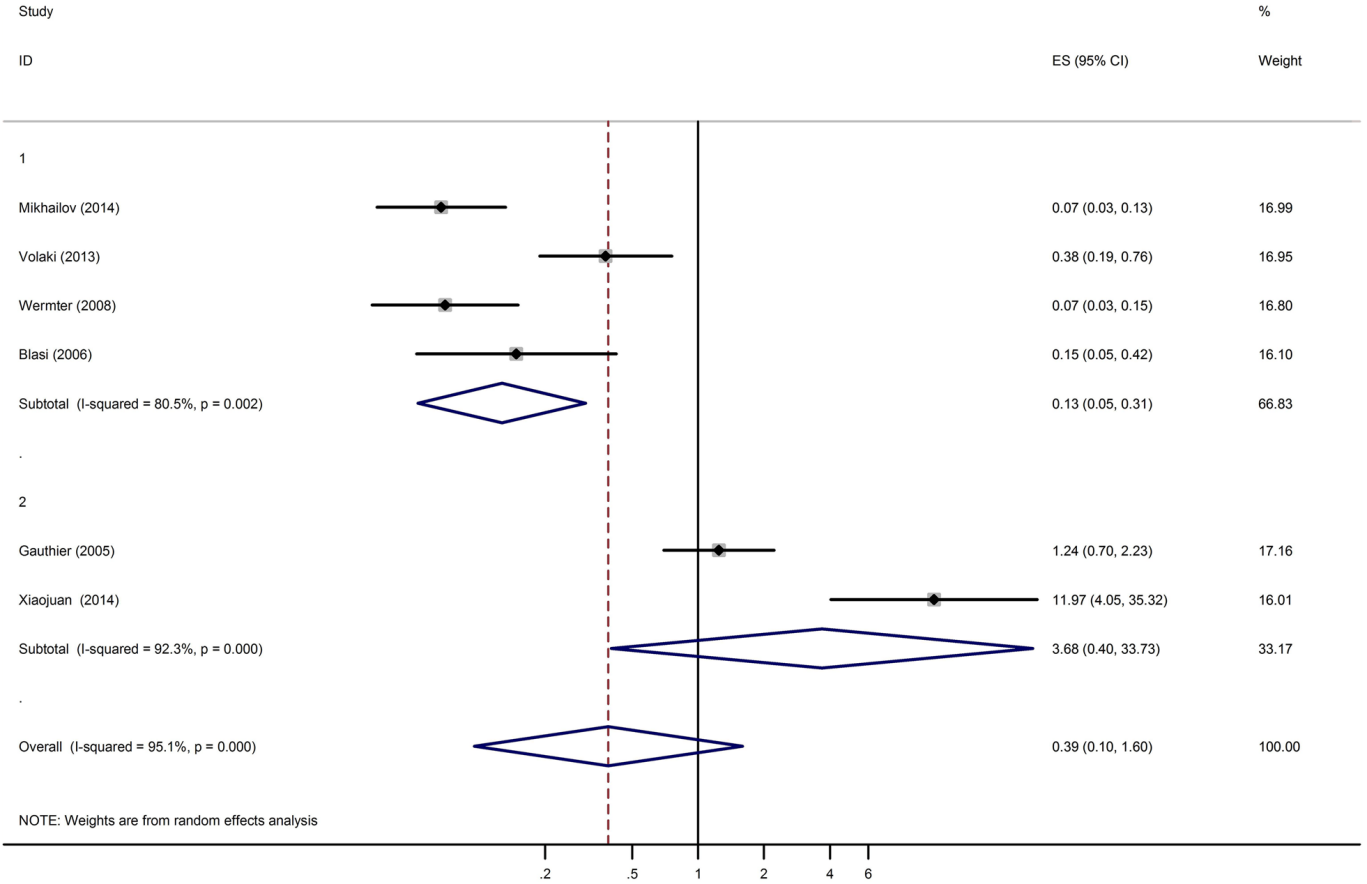
Forest plot for the overall association between the rs3747333, rs3747334 variants and ASD. Each comparison is presented by the surname of the first author and the year of publication

Regarding the rs3747333, rs3747334, no statistically significant correlation with ASD (OR = 0.39, 95% CI 0.10–1.60) was observed under the random-effects model. There was substantial heterogeneity in the estimated effect sizes (*I*-squared: *I*^2^ = 95.1%, *P* = 0.000). We further performed the subgroup analysis by study design and the result showed that there was no statistical significant association existed between the rs3747333, rs3747334 and the risk of ASD under random-effects model (Screening: OR = 0.13, 95% CI 0.05–0.31; Case–control: OR = 3.68, 95% CI 0.40–33.73). There was substantial heterogeneity in the estimated effect sizes (Screening: *I*-squared: *I*^2^ = 80.5%, *P* = 0.002; case–control: *I*-squared: *I*^2^ = 92.3%, *P* = 0.000) (Fig. 2).

### Sensitivity analysis and publication bias

**Fig. 3:**
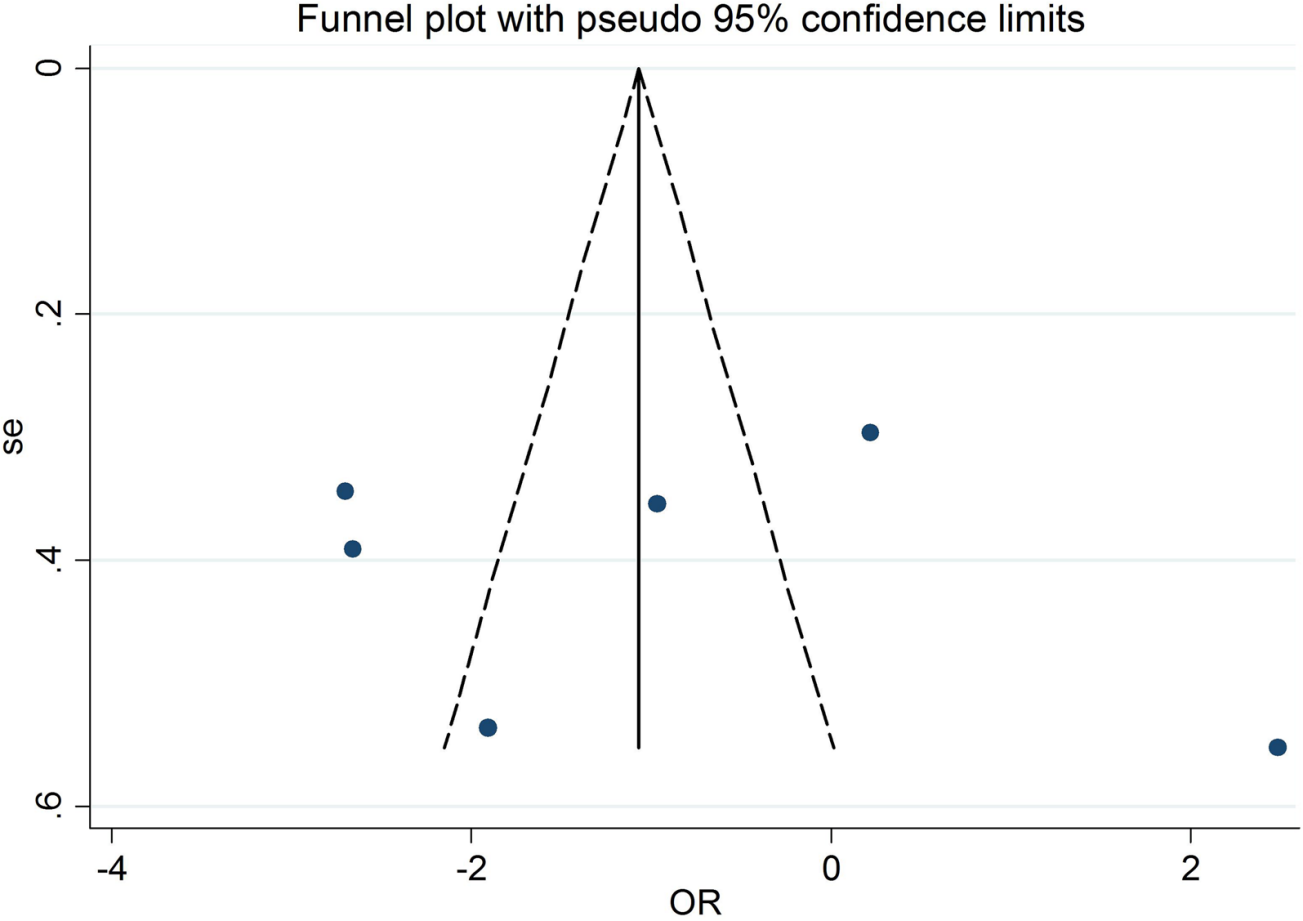
Funnel plots for association studies between the rs3747333, rs3747334 and risk of ASD

The aim of sensitivity analysis was to assess the effects of each study on the pooled OR and ensure that none of solo study was completely responsible for the combined results. The results of sensitivity analysis showed that the pooled OR was not materially affected before and after removal of individual study (see Additional file 1: Figures S1–S6). The studies distribution of the funnel plot was substantially symmetrical about the combined effect size in Fig. 3. Visual inspection of the funnel plot revealed obvious asymmetry.

## Discussion

In the current meta-analysis, pooling the data from 6 published studies indicated the rs3747333 and rs3747334 in NLGN4X were not a risk for ASD. The rs3747333, rs3747334 are linked in exon 6 of NLGN4X, which localized on Xq13.1 and Xp22.33, encode a member of a family of neuronal cell surface proteins serving as splice site specific ligands for neurexins and may be involved in the formation, organization, and remodeling of central nervous system synapses. In our meta-analysis, the most works showed that no evidence for involvement of the rs3747333, rs3747334 in NLGN4X in probands with ASD [9, 11, 14, 15, 18], but Xiaojuan’ work form Chinese sample, conversely, identified that the rs3747333 and rs3747334 in NLGN4X gene significantly associated with the risk for ASD [16]. The inconsistent results of these works are, to a very great extent, determined by ethnicity. However, it is needed to recollect more Chinese sample to identify the risk of the rs3747333, rs3747334 in NLGN4X for ASD.

The main limitation of this meta-analysis is that there was a significant heterogeneity in the estimated effect sizes across studies for the rs3747333, rs3747334 with ASD, suggesting that potential moderator variables, such as ethnicity, may cause the observed differences in effect sizes across studies. Another limitation is that we collected too few studies to reliably test moderators of that heterogeneity, and there may be some potential bias. We performed sensitivity analysis, but the results showed that the pooled OR was not altered materially when ruling out each individual study. A recent meta-analysis using genome-wide association (GWAS) analysis showed that over 16,000 individuals with highlights a novel locus at 10q24.32 and a significant overlap with schizophrenia [19], which made a great contribution to understand the complicated pathogenesis of autism spectrum disorder. Thus, genome-wide association analysis as a better approach to the study of the genetics of ASD should be applied in the future work.

## Conclusions

In summary, our meta-analysis indicates that there was no significant risk elevation between the rs3747333, rs3747334 variants and ASD based on the currently published data. However, the meta-analysis adds new information to the ongoing debate relating to the risk of NLGN4X for ASD. Further investigations of these findings are merited by well-designed and large-sample multi-centric studies.

## Additional file

**Additional file 1.** Supplementary material.

## Data Availability

Not applicable.

## References

[1] DSM-5 American Psychiatric Association (2013). Diagnostic and statistical manual of mental disorders.

[2] Gauthier J, Bonnel A, Stonge J, Karemera L, Laurent S, Mottron L, Fombonne E, Joober R, Rouleau GA (2005). NLGN3/NLGN4 gene mutations are not responsible for autism in the Quebec population. Am J Med Genet Part B Neuropsychiatr Genet.

[3] Xu X, Xiong Z, Zhang L, Liu Y, Lu L, Peng Y, Guo H, Zhao J, Xia K, Hu Z (2014). Variations analysis of NLGN3 and NLGN4X gene in Chinese autism patients. Mol Biol Rep.

[4] Südhof TC (2008). Neuroligins and neurexins link synaptic function to cognitive disease. Nature.

[5] Volaki K, Pampanos A, Kitsioutzeli S, Vrettou C, Oikonomakis V, Sofocleous C, Kanavakis E (2013). Mutation screening in the Greek population and evaluation of NLGN3 and NLGN4X genes causal factors for autism. Psychiatr Genet.

[6] Elsabbagh M, Divan G, Koh YJ, Kim YS, Kauchali S, Marcín C, Montiel-Nava C, Patel V, Paula CS, Wang C (2012). Global prevalence of autism and other pervasive developmental disorders. Autism Res.

[7] Liu Y, Du Y, Liu W, Yang C, Liu Y, Wang H, Gong X (2013). Lack of association between NLGN3, NLGN4, SHANK2 and SHANK3 gene variants and autism spectrum disorder in a Chinese population. PLoS ONE.

[8] Wermter AK, Kamp-Becker I, Strauch K, Schulte-Körne G, Remschmidt H (2008). No evidence for involvement of genetic variants in the X-linked neuroligin genes NLGN3 and NLGN4X in probands with autism spectrum disorder on high functioning level. Am J Med Genet B Neuropsychiatr Genet..

[9] Underwood E. People on autism spectrum die 18 years younger than average. Science. 2016.

[10] Francesca B, Bacchelli E, Pesaresi G, Carone S, Bailey AJ, Maestrini E (2006). Absence of coding mutations in the X-linked genes neuroligin 3 and neuroligin 4 in individuals with autism from the IMGSAC collection. Am J Med Genet Part B Neuropsychiatr Genet.

[11] Gauthier J, Bonnel A, St-Onge J, Karemera L, Laurent S, Mottron L, Fombonne E, Joober R, Rouleau GA (2005). NLGN3/NLGN4 gene mutations are not responsible for autism in the Quebec population. Am J Med Genet B Neuropsychiatr Genet..

[12] Chaste P, Leboyer M (2012). Autism risk factors: genes, environment, and gene-environment interactions. Dialogues Clin Neurosci.

[13] Avdjieva-Tzavella DM, Todorov TP, Todorova AP, Kirov AV, Hadjidekova SP, Rukova BB, Litvinenko IO, Hristova-Naydenova DN, Tincheva RS, Toncheva DI (2012). Analysis of the genes encoding neuroligins NLGN3 and NLGN4 in Bulgarian patients with autism. Genet Couns..

[14] Mikhailov A, Fennell A, Plong-On O, Sripo T, Hansakunachai T, Roongpraiwan R, Sombuntham T, Ruangdaraganon N, Vincent JB, Limprasert P (2014). Screening of NLGN3 and NLGN4X genes in Thai children with autism spectrum disorder. Psychiatr Genet.

[15] Higgins JPT, Green S. Cochrane handbook for systematic reviews of interventions, vol. 5. Wiley Online Library; 2008.

[16] Holmans P (2017). Meta-analysis of GWAS of over 16,000 individuals with autism spectrum disorder highlights a novel locus at 10q24.32 and a significant overlap with schizophrenia. Mol Autism..

[17] Lawson-Yuen A, Saldivar JS, Sommer S, Picker J (2008). Familial deletion within NLGN4 associated with autism and Tourette syndrome. Eur J Hum Genet..

[18] Laumonnier F, Bonnet-Brilhault F, Gomot M, Blanc R, David A, Moizard MP, Raynaud M, Ronce N, Lemonnier E, Calvas P, Laudier B, Chelly J, Fryns JP, Ropers HH, Hamel BC, Andres C, Barthelemy C, Moraine C, Briault S (2004). X-linked mental retardation and autism are associated with a mutation in the NLGN4 gene, a member of the neuroligin family. Am J Hum Genet..

[19] Blasi F, Bacchelli E, Pesaresi G, Carone S, Bailey AJ, Maestrini E (2006). Absence of coding mutations in the X-linked genes neuroligin 3 and neuroligin 4 in individuals with autism from the IMGSAC collection. Am J Med Genet B Neuropsychiatr Genet..

